# Safety of indocyanine green use in bariatric and metabolic surgery

**DOI:** 10.20452/wiitm.2025.17964

**Published:** 2025-07-04

**Authors:** Mateusz Wityk, Maciej Bobowicz, Natalia Dowgiałło‑Gornowicz

**Affiliations:** Department of General and Oncological Surgery, Regional Health Centre, Lubin, Poland; Department of Radiology, Medical University of Gdansk, Gdańsk, Poland; Department of General, Minimally Invasive and Elderly Surgery, Collegium Medicum, University of Warmia and Mazury, Olsztyn, Poland

**Keywords:** bariatric surgery, fluorescence, indocyanine green, metabolic surgery, obesity

## Abstract

**INTRODUCTION:**

Intraoperative indocyanine green (ICG) is increasingly used in surgery. In metabolic and bariatric surgery, it allows visualization of the tissues and blood supply to anastomoses in real time.

**AIM:**

The objective of this prospective cohort study was to evaluate the safety and efficacy of ICG usage in metabolic and bariatric surgery.

**MATERIALS AND METHODS:**

The study was conducted from July 2022 to December 2023. A total of 171 metabolic and bariatric procedures with ICG perfusion tests were performed, comprising 93 sleeve gastrectomies, 17 one-anastomosis gastric bypasses, 51 Roux-en-Y gastric bypasses, 7 primary single anastomosis duodeno-ileal bypasses with sleeve gastrectomies, and 3 redo surgeries.

**RESULTS:**

Out of 171 patients included in the study, 143 (83.6%) were women. Mean (SD) age of the participants was 41.8 (9.7) years, and mean (SD) body mass index was 44.6 (6.3) kg/m^2^. Mean (SD) operation time was 92.9 (47.7) minutes, and mean (SD) length of hospital stay was 2.1 (0.8) days. Surgical complications occurred in 4 patients (2.3%). No local or general adverse symptoms or complications, including allergic reactions, were observed after intravenous administration of ICG. The patients’ allergies did not influence adverse events related to the administration of the dye.

**CONCLUSIONS:**

The use of intraoperative ICG in metabolic and bariatric surgery is safe, and adverse events and dye-related complications are very rare.

## INTRODUCTION

Indocyanine green (ICG) utilization in surgery increases annually with a broad adoption of new applications. In addition to visualization of the tissues and anastomosis blood supply, it is also used to detect lymph nodes and lymphatic vessels, primary tumors, and metastases. It is instrumental in trauma surgery, microsurgery, and burns treatment.^1^^,^^2^^,^^3^^,^^4^^,^^5^^,^^6^^,^^7^^,^^8^^,^^9^^,^^10^ ICG is a water-soluble, anionic dye compatible with plasma proteins found in the bloodstream. Half-life of the dye is short and ranges from 150 to 180 seconds, depending on the efficiency of liver function. In the liver, ICG is absorbed from the bloodstream in less than 20 minutes after intravenous injection and excreted into bile without undergoing metabolic changes.^11^^,^^12^^,^^13^

ICG was first introduced in clinical practice at the Mayo Clinic in 1955, and gained significant attention for its diagnostic potential. Four years later, it received approval from the United States Food and Drug Administration for various medical applications. Its ability to highlight vascular structures and tissues using near-infrared fluorescence has made it an invaluable tool.^8^**^,^**^14^

Despite nearly 70 years of using ICG in medicine, there is still a need for more prospective, large-population randomized studies in metabolic and bariatric surgery (MBS). This prospective study aimed to evaluate the safety of using ICG in MBS, which may contribute to its increased use in this field.

## AIM

The primary objective of this study was to examine the incidence of allergic reactions induced by ICG. The secondary aim was to evaluate mortality and morbidity rates during the early postoperative period.

## MATERIALS AND METHODS

### Patients

This prospective observational cohort study was conducted in patients who underwent MBS from July 2022 to December 2023. Inclusion criteria comprised age over 18 years and meeting the qualification criteria for any MBS procedure.^15^ Exclusion criteria were: age below 18 years, inability to express informed consent, lack of consent to participate in the study, and other than MBS surgical procedure. Of 318 eligible patients, 171 agreed to participate in the study.

**FIGURE 1:**
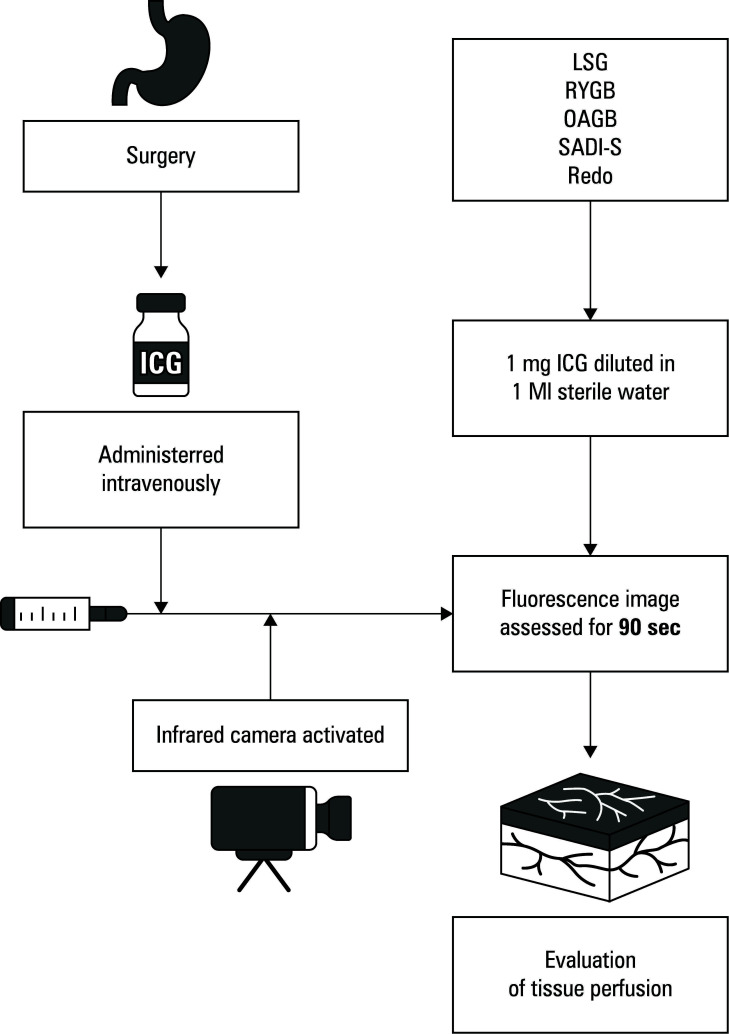
Technique of intraoperative indocyanine green administration

Abbreviations: ICG, intraoperative indocyanine green; LSG, laparoscopic sleeve gastrectomy; OAGB, one-anastomosis gastric bypass; RYGB, Roux -en -Y gastric bypass; SADI -S, single anastomosis duodeno -ileal bypass with sleeve gastrectomy

**TABLE 1:** Characteristics of the patients

Parameter	Value
Men, n (%)	28 (16.4)
Age, y, mean (SD)	41.8 (9.8)
Preoperative BMI, kg/m², median (IQR)	43.7 (40.8–49.5)
Length of stay, d, mean (SD)	2.1 (0.8)
Operative time, min, median (IQR)	80 (60–115)
Type 2 diabetes, n (%)	42 (24.6)
Hypertension, n (%)	71 (41.5)

Abbreviations: BMI, body mass index; IQR, interquartile range

All patients were managed according to the Enhanced Recovery After Surgery guidelines.^16^ Each patient was given a carbohydrate-rich drink 2 hours before surgery to help maintain energy levels and minimize the risk of fasting-related complications. Pharmacological and mechanical deep vein thrombosis prophylaxis was applied. Standard of care did not involve placement of a peritoneal drain or urinary catheter. Routine postoperative laboratory blood tests were not conducted. Each patient was qualified for early physical rehabilitation within the first hour following surgery, and an oral liquid diet was introduced. The discharge protocol contained effective pain management, oral diet tolerance, proper mobilization and normal-range heart rate, blood pressure, and oxygen saturation levels.

All patients were carefully observed during hospitalization. Surgical evaluation was performed during outpatient visits 10 days and 6 months after surgery. Data on weight loss, possible allergic symptoms, medications taken, and postoperative complications were collected during follow-up. Additionally, the patients were enrolled in the hospital-at-home discharge program with close monitoring by a bariatric nurse every 3 months, and 24/7 over-the-phone teleconsultations in the event of disturbing symptoms. The complications were assessed according to the Clavien–Dindo classification.^17^

### Surgical technique

The same surgical team performed all procedures with standardized patient placement in the beach-chair position, 12 mm Hg pneumoperitoneum, and the same surgical tools. A 34 French bougie was utilized to measure the sleeve and gastric pouch during one-anastomosis gastric bypass (OAGB) and Roux-en-Y gastric bypass (RYGB). There was no additional reinforcement of the staple line. We used 150 cm of biliopancreatic limb for OAGB and RYGB. The standard alimentary limb length for RYGB was 75 cm. In the case of single anastomosis duodeno-ileal bypass with sleeve gastrectomy (SADI-S), the length of the common limb was 275 cm. Anastomoses in the case of OAGB and RYGB were performed using a stapler, with the closure of the common channel using 2 layers of barbed suture. The duodeno-ileal anastomosis was hand-sewn in single anastomosis. In all OAGB and RYGB cases, the mesenteric gap and Petersen space were closed with nonabsorbable sutures. A methylene blue leak test was performed in all procedures following the local MBS safety protocol.

### Indocyanine green administration

ICG was used during surgery as an adjunct procedure to detect any ischemic foci. Special attention was paid to the parts of the digestive tract undergoing surgery, that is, the gastric sleeve and the gastrointestinal, entero-intestinal, and duodeno-intestinal anastomoses. The fluorescence assessment protocol was based on literature research findings and our previous experience with ICG use. Following the initial procedural steps, 1 mg of ICG was diluted in 1 ml of sterile water, and administered intravenously via a catheter inserted in the left antecubital fossa. Then, 10 ml of 0.9% sodium chloride solution was administered through the same venous port. The 30-degree infrared 1588 AIM + SPY Fluorescence Technology camera (Stryker, Kalamazoo, Michigan, United States) was then activated to enable real-time blood flow screening. The fluorescence image was assessed for at least 90 seconds after the dye became visible in the tissues. The surgical team meticulously evaluated tissue perfusion levels. In the cases of identifying ischemia or inadequate perfusion, the surgical plan was promptly adjusted to prevent postoperative complications (FIGURE 1).

### Statistical analysis

A descriptive statistical analysis was conducted with Statistica software 13.PL (Tibco Software Inc., Palo Alto, California, United States). The normal distribution was checked using the Shapiro–Wilk test. Numbers and percentages were used for numerical variables. For continuous variables, mean (SD) or median (interquartile range [IQR]) were used when appropriate.

### Ethics

The study was conducted in accordance with the ethical standards of the 1964 Declaration of Helsinki and its subsequent amendments. The study protocol was approved by the Bioethics Committee of Gdansk Medical Chamber (KB-33/24.)

## RESULTS

A total of 171 patients were included in the study. Of them, 83.6% were women. Mean (SD) age of the participants was 41.8 (9.8) years. Median (IQR) body mass index was 43.7 (40.8–49.5) kg/m^2^.

As many as 41.5% of the patients suffered from hypertension and 24.6%, from type 2 diabetes (TABLE 1).

In the analyzed group, 27 patients (15.8%) had allergies to pharmaceuticals and inhalants. Sixteen patients (9.4%) reported being allergic to antibiotics, 6 (3.5%), to pollen, 2 (1.2%), to metamizole, and 2 (1.2%), to nonsteroidal anti-inflammatory drugs. One participant (0.6%) was allergic to iodine, 1 (0.6%), to tramadol, and 1 (0.6%), to salicylates. Nine patients (5.3%) had asthma. The iodine allergy was identified during follow-up when treating a wound unrelated to MBS. Despite previously undiagnosed iodine allergy, the intraoperative use of ICG increases the likelihood of an allergic reaction.

A total of 171 MBSs with ICG perfusion tests were performed, comprising 93 sleeve gastrectomies (SGs), 17 OAGBs, 51 RYGBs, 7 primary SADI-Ss, and 3 redo surgeries converting SG to SADI-S. Median (IQR) operation time was 80 (60–115) minutes, and mean (SD) length of hospital stay was 2.1 (0.8) days.

### Ischemia

All patients undergoing SG and SADI-S had proper blood flow confirmed with fluorescence. In OAGB, 1 ischemia of the gastrointestinal anastomosis, and in RYGB, 1 ischemia of the terminal part of the enzymatic loop were detected. In the case of OAGB, ischemia was not visible prior to ICG administration. In RYGB, before ICG administration, the loop appeared darker, and ICG administration showed improper blood flow. The surgical strategy was changed intraoperatively in both cases, and the postoperative course was uneventful. Before the incorporation of ICG into surgical practice, the leakage rate in MBS in our center was 0.4%.

### Safety profile

In the study group, no local or general adverse symptoms or complications, including allergic reactions, were observed after intravenous administration of ICG. The complications that occurred in the perioperative period were not related to fluorescence. The patients’ allergies did not influence the occurrence of adverse events related to the administration of ICG.

### Complications

Complications occurred in 4 patients (2.3%). There were 2 cases (1.2%) of gastrointestinal bleeding, 1 after SG from the gastric sleeve staple line and another after RYGB from the gastrojejunal anastomosis staple line. Both cases required endoscopic intervention and were successfully managed, classified as Clavien–Dindo class 3a complications. Hospitalization was extended to 3 days after SG and 4 days after RYGB. In 1 SG patient (0.6%), the small intestine was perforated during the creation of pneumoperitoneum with an optical trocar. The perforation was detected on the second postoperative day. The patient required a revisional surgery (Clavien–Dindo class 3b) and a hospital stay of 12 days. In 1 patient (0.6%) undergoing SADI-S, the duodenum was ruptured by a barbed suture, causing leakage of methylene blue and the inability to suture the perforation. Intraoperatively, the decision was made to convert to OAGB (Clavien–Dindo class 3b). Hospitalization lasted 3 days. Posthospitalization period was uneventful in all cases.

## DISCUSSION

Among 171 MBSs performed using ICG, no early or long-term complications related to intravenous administration of the dye were observed. One miligram of ICG was sufficient in all cases to assess tissue blood flow. Fluorescence proved helpful in evaluating ischemia. It allowed the surgeons to intraoperatively modify the procedure and avoid 2 severe OAGB and RYGB procedure complications. The complications that actually occurred were not related to the administration of ICG. The study population was heterogeneous, which enabled us to assess the safety of fluorescence in all types of primary operations performed. The results seem to be relevant for ICG use in MBS. To our knowledge, this is the first study assessing the safety of ICG in these types of procedures.

The application of ICG is becoming increasingly prevalent across various medical disciplines.^1^^,^^2^^,^^3^^,^^4^^,^^5^^,^^6^^,^^7^^,^^8^ New opportunities for using ICG in surgical procedures continue to emerge each year. Fluorescence imaging, facilitated by ICG, is employed to evaluate adequate blood perfusion to organs or gastrointestinal anastomoses, and proves valuable in identifying primary tumors, lymph nodes, or metastases within parenchymal organs.^9^^,^^10^ Additionally, ICG is used for mapping the biliary tree, ureters, and thoracic duct, significantly lowering the risk of injury to these structures and minimizing associated complications.^18^^,^^19^^,^^21^^,^^22^ The use of ICG has expanded into emergency surgery, where it aids in the evaluation of post-traumatic changes and revascularization following vascular injuries, and it plays an essential role in burn management and reconstructive surgery.^2^^,^^3^^,^^4^^,^^5^^,^^6^^,^^7^**^,^**^23^ As the capabilities of ICG continue to evolve, its role in diagnostic and therapeutic interventions is expected to grow, further enhancing surgical precision and patient outcomes.

ICG in MBS has become an essential adjunct in evaluating tissue perfusion and identifying potential areas of ischemia. While the overall risk of compromised blood flow during these procedures is relatively low, ICG fluorescence imaging is a valuable tool for early detection of any abnormalities in tissue vascularization, which can help prevent ischemic complications.^24^^,^^25^^,^^26^ In addition, ICG fluorescence is also helpful in assessing the integrity of anastomoses, providing real-time visualization that can detect leaks.^27^**^,^**^28^ Furthermore, fluorescence is utilized as a marker to identify the source of bleeding in the gastrointestinal tract.^29^ Despite its growing use and potential benefits, the data on ICG fluorescence imaging in surgical treatment of obesity are still limited.^30^^,^^31^^,^^32^^,^^33^^,^^34^^,^^35^

The use of ICG is safe, and complications are infrequent. Severe adverse reactions occur in 0.05% of cases. In the available literature, there are 2 case reports of anaphylactic shock after intravenous ICG administration.^36^^,^^37^^,^^38^ The mechanism of the allergic reaction is not fully understood, but the proposed theory includes immunoglobulin E-mediated hypersensitivity, nonallergic histamine release, complement activation, and release of other inflammatory mediators. Chronic renal impairment predisposes to adverse effects.^9^**^,^**^38^^,^^39^^,^^40^

Some of the risk factors for an anaphylactic reaction include a history of allergic diseases, such as asthma, food allergy, or drug allergy. Additionally, hypertension and type 2 diabetes may increase this risk.^38^ In the observed group, the occurrence of inhalant, food, and drug allergies or asthma did not induce allergy to ICG.

The major limitations of the study are its single-center character and a relatively small, heterogeneous study group. The very low frequency of adverse events after ICG administration can introduce some bias. Severe complications may affect 0.05% of patients after dye administration, and further reports on larger cohorts are needed to reach final conclusions.^8^**^,^**^38^

## CONCLUSIONS

This is the first publication assessing the safety of using ICG in MBS. ICG appears to be associated with a low risk of allergic complications, and its use may be beneficial in reducing ischemic complications. Given the exceedingly rare occurrence of allergic reactions following ICG administration, a comparative study with sufficient power would be necessary to reach definitive conclusions.

## JOURNAL INFORMATION

Videosurgery and Other Miniinvasive Techniques is an official journal of the Videosurgery Foundation.
